# The representation of sediment source group tracer distributions in Monte Carlo uncertainty routines for fingerprinting: An analysis of accuracy and precision using data for four contrasting catchments

**DOI:** 10.1002/hyp.13736

**Published:** 2020-03-10

**Authors:** Simon Pulley, Adrian L. Collins, J. Patrick Laceby

**Affiliations:** ^1^ Sustainable Agriculture Sciences Rothamsted Research Devon UK; ^2^ Environmental Monitoring and Science Division, Alberta Environment and Parks Calgary Alberta Canada

**Keywords:** accuracy, Monte Carlo simulation, precision, sediment fingerprinting, sediment sources, sediment tracing

## Abstract

Previous studies comparing sediment fingerprinting un‐mixing models report large differences in their accuracy. The representation of tracer concentrations in source groups is perhaps the largest difference between published studies. However, the importance of decisions concerning the representation of tracer distributions has not been explored explicitly. Accordingly, potential sediment sources in four contrasting catchments were intensively sampled. Virtual sample mixtures were formed using between 10 and 100% of the retrieved samples to simulate sediment mobilization and delivery from subsections of each catchment. Source apportionment used models with a transformed multivariate normal distribution, normal distribution, 25th–75th percentile distribution and a distribution replicating the retrieved source samples. The accuracy and precision of model results were quantified and the reasons for differences were investigated. The 25th–75th percentile distribution produced the lowest mean inaccuracy (8.8%) and imprecision (8.5%), with the Sample Based distribution being next best (11.5%; 9.3%). The transformed multivariate (16.9%; 17.3%) and untransformed normal distributions (16.3%; 20.8%) performed poorly. When only a small proportion of the source samples formed the virtual mixtures, accuracy decreased with the 25th–75th percentile and Sample Based distributions so that when <20% of source samples were used, the actual mixture composition infrequently fell outside of the range of uncertainty shown in un‐mixing model outputs. Poor performance was due to combined random Monte Carlo numbers generated for all tracers not being viable for the retrieved source samples. Trialling the use of a 25th–75th percentile distribution alongside alternatives may result in significant improvements in both accuracy and precision of fingerprinting estimates, evaluated using virtual mixtures. Caution should be exercised when using a normal type distribution, without exploration of alternatives, as un‐mixing model performance may be unacceptably poor.

## Graphical abstract

The representation of source group tracer concentrations is perhaps the largest difference between sediment fingerprinting un‐mixing models. Despite this, the effects of different distributions on model accuracy have not been explored explicitly. ‘This study compared a transformed multivariate normal, a normal and a 25th–75th percentile distribution as well as a distribution replicating the retrieved source samples. The 25th–75th percentile distribution produced the lowest mean inaccuracy (8.8%), with the Sample Based being next best (11.5%). The transformed multivariate (16.9%) and untransformed normal distributions (16.3%) performed poorly.

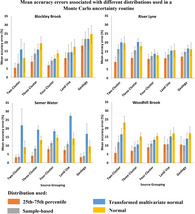

## 1 INTRODUCTION

Elevated fine‐grained sediment mobilization and delivery degrades water quality and aquatic ecology and increases the costs of potable water treatment (Berry, Rubinstein, Melzian, & Hill, 2003; Bilotta & Brazier, 2008; Collins et al., 2011; Jones, Collins, Naden, & Sear, 2012; Jones, Murphy, Collins, Sear, & Naden, 2012; Kemp, Sear, Collins, Naden, & Jones, 2011; McDonald, Weber, Padowski, Boucher, & Shemie, 2016). Effective management strategies therefore require robust evidence on the nature and distribution of, and relative contributions from, the principal sediment sources in river catchments (Minella, Walling, & Merten, 2014). Here, existing methods for documenting catchment sediment sources comprise both direct and indirect approaches (Collins & Walling, 2004; Loughran & Campbell, 1995). Sediment source fingerprinting, a direct approach to confirming sediment sources, is now increasingly seen as a globally applicable tool (Krishnappan, Chambers, Benoy, & Culp, 2009; Miller, Mackin, & Orbock Miller, 2015; Owens et al., 2016; Walling, 2013).

Pioneering sediment fingerprinting work was founded on qualitative comparisons of source material and target sediment samples to infer sediment sources (Klages & Hsieh, 1975; Wall & Wilding, 1976), but from the 1980s and 1990s, mass balance un‐mixing models became the accepted means of estimating source contributions quantitatively (Collins, Walling, & Leeks, 1997; He & Owens, 1995; Walden, Slattery, & Burt, 1997; Walling & Woodward, 1995; Walling, Woodward, & Nicholas, 1993; Yu & Oldfield, 1989). When apportioning sediment provenance using un‐mixing models, most of the early quantitative sediment source fingerprinting studies represented tracer concentrations using a single mean or median value for each source group (Collins et al., 1997; Walling & Woodward, 1992; Walling & Woodward, 1995). At this time, there was only a limited assessment of the uncertainties associated with the within‐source group variability in source tracer concentrations. The introduction of Monte Carlo uncertainty routines into sediment source fingerprinting methodologies by Franks and Rowan (2000) and Rowan, Goodwill, and Franks (2000) allowed for these uncertainties to be expressed explicitly in modelled outputs and the inclusion of uncertainty routines has since become the norm in robust source fingerprinting studies (Collins et al., 2017; Walling, 2005, 2013). To date, numerous methods of representing the distributions of tracers within sampled sediment source groups have been used in Monte Carlo uncertainty routines. For example, Motha, Wallbrink, Hairsine, and Grayson (2003) and Collins, Walling, Webb, and King (2010) used source group means and standard deviations and this approach remains widely used in international literature (e.g. Aliyanta & Sidauruk, 2019; Brosinsky, Foerster, Segl, & Kaufmann, 2014; Chen, Fang, & Shi, 2016; Dahmardeh Behrooz, Gholami, Telfer, Jansen, & Fathabadi, 2019; Gateuille et al., 2019). Krause, Franks, Kalma, Loughran, and Rowan (2003), Wilkinson et al. (2009); Wilkinson, Hancock, Bartley, Hawdon, and Keen (2013), Haddadchi, Olley, and Pietsch (2015, 2016), Laceby and Olley (2015) and Palazón et al. (2016) all used a Student's *t*‐distribution which gave more weighting to the tails of the distribution than a normal distribution and was considered more appropriate when sample numbers were low. Non‐parametric estimators of location and scale such as median and median absolute deviation (MAD) or either Qn (Collins, Walling, et al., 2010) or Sn (Collins, Zhang, Walling, & Black, 2010) and the 25th–75th percentile inter‐quartile range have also been used (Pulley, Foster, & Antunes, 2015). Qn and Sn are alternative more efficient scale estimates to the MAD and not slanted towards symmetric distributions (Rousseeuw & Croux, 1993). In some studies, distributions have been constructed and repeat sampled for both source group samples and target sediment samples during un‐mixing model uncertainty routines (Collins, Walling, Webb, & King, 2010) with the sampling frequently using Latin Hypercube routines for efficiency and effective sampling of deviate tracer values (Collins, Zhang, Walling, et al., 2012; Collins, Zhang, Walling, Grenfell, & Smith, 2010). Here, the 25th–75th percentile range has the advantage that the distribution of tracers either side of the median does not need to be symmetrical. An alternative approach is to preserve the distribution provided by the tracer analyses on the samples collected to characterize any given source sampled in the study catchment in question, without using an estimator of scale (e.g. Rousseeuw & Croux, 1993) for the distribution (Olley, Brooks, Spencer, Pietsch, & Borombovits, 2013; Pulley & Collins, 2018a).

Over the past 20 years, studies adopting un‐mixing models in sediment fingerprinting studies have primarily used frequentist approaches based on maximum likelihood estimation (Davis & Fox, 2009; Walling, 2005; Walling, Collins, Jones, Leeks, & Old, 2006; Walling, Collins, & Stroud, 2008; Haddadchi, Ryder, Evrard, & Olley, 2013; Owens et al., 2016; Smith, Karam, & Lennard, 2018; Batista et al., 2019). More recently, however, Bayesian un‐mixing models have been experiencing rapid uptake for sediment source fingerprinting purposes and offer potential advantages over frequentist models such as the ability to use informative priors and to include the uncertainty derived from an imperfect knowledge of factors such as the mean, variance and distribution of variables (Davies, Olley, Hawker, & McBroom, 2018; O'hagan & Luce, 2003). Many Bayesian models, such as MixSIAR, assume a normal distribution of tracers within potential sediment sources with the mean and standard deviation values for each source group used as inputs (Gateuille et al., 2019; Stock et al., 2018). A Bayesian model presented by Cooper, Krueger, Hiscock, and Rawlins (2015) formed a multivariate normal distribution to represent the sources within the model; this distribution maintains any correlations between tracers which are present in the retrieved source samples (Cooper, Krueger, Hiscock, & Rawlins, 2014). It is, however, often found that tracer concentrations in sediment source groups are not normally distributed (Collins, Zhang, McChesney, et al., 2012; Collins et al., 2013; Laceby, Huon, Onda, Vaury, & Evrard, 2016; Olley et al., 2013) which represents a major potential source of uncertainty when a normal distribution is used. To address the potential non‐normality of source groups, tracer concentrations are often transformed. For example, Batista et al. (2019) log‐transformed the tracer concentrations before forming the multivariate roughly normal distributions and then back‐transformed using an exponential function during the un‐mixing model Monte Carlo simulations.

A number of studies have compared the errors associated with different un‐mixing model structures by apportioning the sources of artificial and virtual mixtures (Haddadchi, Ryder, Evrard, & Olley, 2014; Palazón et al., 2015), yet limited explanations have been presented as to why some model structures deliver more accurate results than others. Cooper et al. (2014) found that changes to model configuration such as the covariance structure used could exert a significant effect on the results produced. Comparisons can also be complicated by the use of conventional correction factors for particle size and organic matter (e.g. Walling et al., 2006, 2008) in some procedures which can introduce significant uncertainties (Smith & Blake, 2014), meaning that their application must be assessed on a sample by sample basis (Collins, Walling, et al., 2010). In addition, Laceby and Olley (2015) used artificial source mixtures to show that tracer weightings can potentially decrease model accuracy. Overall, although un‐mixing model structures can include a variety of corrections and weightings (Collins et al., 2017; Collins, Walling, et al., 2010; Walling, 2005) one of the most important differences between un‐mixing model structures concerns how the distributions of tracer concentrations in the sampled source groups are represented.

When assessing which tracer distribution is likely to be optimal for use, a key consideration is whether it is representative of tracer concentrations present in catchment source groups. For example, using an un‐transformed normal distribution, when tracer concentrations in catchment sources are not normally distributed, is likely to result in source apportionment uncertainties which are unaccounted for the Monte Carlo analysis. It is also often the case that the time and budgetary resources of a study will limit the number of source samples which can be retrieved and analysed, in turn, potentially limiting the accuracy of the tracer distributions used as input for the un‐mixing model. A second major consideration here is that erosion and sediment delivery are highly unlikely to be uniform throughout a catchment and are likely to vary spatially and temporally depending on hydrological conditions and slope‐to‐channel connectivity (Bracken, Turnbull, Wainwright, & Bogaart, 2015; Fryirs, 2013). Therefore, even with an unlimited number of source samples retrieved from a catchment and their perfect representation within a Monte Carlo routine, the sources of a specific sediment sample will likely not follow a tracer distribution representative of concentrations present in entire catchment‐wide source groups. As a result, it is almost inevitable that the source group tracer distributions used in an un‐mixing model will not be ideally suited to each target sediment sample being fingerprinted. It is, however, little understood what effect this will have on results and which type of distribution will have the most accurate results when accounting for discrepancies between the tracer distributions present in a catchment and those actually incorporated into the un‐mixing model structure.

There are a number of potential advantages to the different distributions available for modelling. Pulley et al. (2015) showed that a large contrast in tracer concentrations between sources and low within‐source group variability was essential for minimizing uncertainty in un‐mixing model outputs. Therefore, using a tracer distribution with as narrow a range of values as possible, such as the 25th–75th percentile range, will likely result in a lower uncertainty in the model outputs. However, given that the mobilization and delivery of sediment from individual sources is unlikely to be uniform throughout the study catchment, meaning that highly localized sediment inputs are a distinct possibility in some if not many storm events, there is a significant risk that the actual sediment provenance could fall outside of the uncertainty range produced by the un‐mixing model if too narrow a tracer distribution is imputed into the model. Owing to the high labour and financial costs of source material sample collection, preparation and analysis, most studies are limited in the number of source samples which can be analysed and therefore use an assumed normal distribution. Here, however, the presence of outliers with very high or low tracer concentrations will likely cause a large range of values to be generated in the Monte Carlo routine, resulting in a significant increase in the uncertainty for modelled source apportionment. Due to this risk, outliers have been removed as part of some sediment fingerprinting procedures (e.g. Gellis et al., 2016) although a judgement must clearly be made as to which samples are classified as outliers, and this may become increasingly difficult when only a small number of samples are retrieved for each source group included in the catchment sampling strategy. This approach also forms a symmetrical distribution either side of the mean which may not accurately represent what is found in the catchment. The approach of using a distribution matching that of the sampled sources (Olley et al., 2013; Pulley & Collins, 2018a), without applying any estimators of location or scale, appears to be a more robust solution since it is not as affected by outliers and can be a‐symmetrical. A downside, however, is that it does require very thorough source sampling and the analysis of large numbers of samples to ensure that the distributions used are truly representative of natural tracer variability across space.

The above background clearly underscores a gap in existing international literature meaning there is need for explicit consideration of the impact on un‐mixing model accuracy and precision, of different options for constructing tracer distributions used as inputs. Accordingly, our overarching aim was to understand how different tracer distributions imputed into in a frequentist un‐mixing model, with an uncertainty routine, affect the accuracy and precision of the results. In addressing this aim, we compared un‐mixing model performance using a transformed multivariate normal distribution (TMV Normal), an untransformed non‐multivariate normal distribution (Normal), a 25th–75th percentile distribution (25th‐75th) and a sample based distribution (Sample Based). Virtual mixtures of the potential sediment sources in four study areas were formed using subsets of the source sample datasets with between 10 and 100% of the source sampled included in each mixture. These mixtures were aimed at simulating the effects of sediment mobilization and delivery from only a small proportion of the area within each catchment which will cause a mismatch between the tracer distribution incorporated into the un‐mixing model and that of the target sediment mixture being fingerprinted. More specifically, data from four intensively sampled catchments were utilized for this analysis with three different tracer types and multiple different source group classifications and composite fingerprints. Importantly, the scope of this study is related to the uncertainties associated with un‐mixing modelling arising from the choice of tracer distributions for end members and does not extend to incorporating the uncertainties associated with sampling, sample processing, tracer analysis or non‐conservatism.

## 2 STUDY SITES

**Figure 1:**
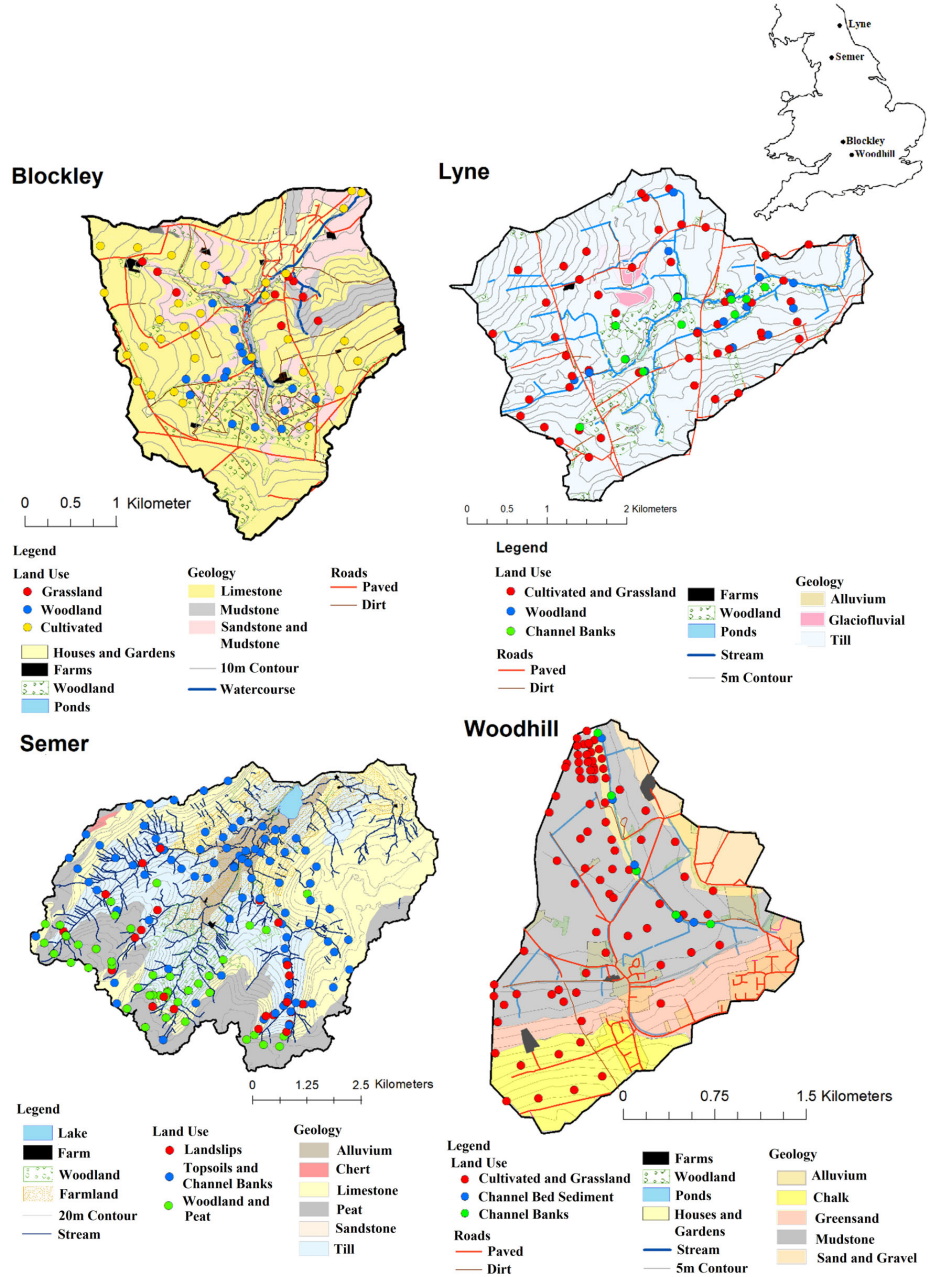
The four study catchments showing geology and source sampling locations with their associated land uses. Note that the land uses shown are the simplified groups used in the sediment source fingerprinting exercise

Four river catchments (Figure 1) in different parts of the United Kingdom were selected for this study. These catchments have different land uses and geologies (Table 1) requiring a different basis for source discrimination using the trialled tracers. The catchment of Blockley Brook (8.52 km^2^) is located at the village of Blockley in the Cotswold district of Gloucestershire. It is characterized by a series of shallow ditches and lakes forming most of the drainage network. Defined channels with distinct channel banks are only present adjacent to and downstream of Blockley village. Land use is a mix between woodland in the centre of the catchment and cultivated fields primarily used for cereals and legumes in the rest of the catchment. There is a small area of grassland in the form of a formal lawn in the north west of the catchment and areas of sheep and cattle grazing close to the village. The village of Blockley covers much of the lower catchment; however, the river channel does not flow directly through built up areas significantly limiting the potential for sediment inputs from urban road dusts which were therefore not considered as a source for this particular study. The river channel backs onto gardens within the village providing little opportunity for urban road dusts to contribute to the sediment load. Catchment geology is composed primarily of limestone in the upper catchment, with mudstones and mixed sand and mudstones in the lower catchment.

The upper part of the River Lyne catchment (11.70 km^2^) selected for this study is located in North West England close to the village of Tritlington (Figure 1). Its land use is a mixture of cattle and sheep grazing and cultivation for wheat. There is a pattern of cultivation dominating in the eastern lower catchment and grassland in the western upper catchment. Catchment geology is almost entirely composed of uniform glacial till, with some small alluvial and glaciofluvial deposits present. Channel banks are up to 2.5 m in height with clear evidence of heavy cattle poaching of channel margins in the lower catchment.

The catchment of the lake, Semer Water (43.05 km^2^), is in the Yorkshire Dales national park close to the village of Bainbridge (Figure 1). The lake is 246 m above sea level and the watershed is a maximum of 700 m, forming a steeply sloping catchment. Local geology is composed of alluvium in the lower catchment close to the lake, diamicton in valley bottoms, limestone on hillslopes and blanket peat along the watershed. The alluvial deposits in the valley bottom close to the lake are heavily waterlogged and form a wetland at the inlet to the lake. Land use is rough grazing by sheep throughout most of the catchment, but with more intensive sheep and cattle grazing in the lower areas outside of the wetland. There is an area of commercial forestry in the upper centre portion of the catchment. Channel banks are generally very shallow (<20 cm) and formed primarily of rock; the exception being some >2 m high banks in the wetland directly upstream of the lake itself. Landslips are present throughout steeper sloping areas over the diamicton geology, exposing the underlying material and creating highly erodible deposits.

The Woodhill Brook catchment (4.4 km^2^) is located close to the village of East Challow in Oxfordshire (Figure 1). Land use is dominated by wheat and barley cultivation, with some small fields used for light grazing or hay production present in the upper catchment adjacent to the village and overlying the greensand geology. The geology in the remainder of the catchment is mudstones in the lower portion and an outcrop of limestone at the highest ground in the south west. The channel beds of the stream consist of an up to 30 cm deep layer of thick anoxic mud which was considered to be a sediment source, rather than a sink, due to the very large quantities of readily mobilized material present. Channel banks were generally shallow (<30 cm) and do not appear to be experiencing significant erosion in most locations. A narrow corridor of woodland (<10 m diameter) separates cultivated land from the river channel.

## 3 MATERIALS AND METHODS

### 3.1 Field sampling

In each catchment, efforts were made to achieve a high source sampling density so that the sampled distributions of tracer concentrations were representative of those present within the study areas. Adequate sample numbers (Table S1) were also retrieved to create virtual mixtures using the subsamples from each source dataset. Samples of topsoils susceptible to erosion and sediment mobilization were retrieved as a composite of five subsamples from within 5 m of each individual sampling point. The samples were retrieved from the top 2–3 cm of the soil profile as this is the depth to which the most widespread erosion processes (i.e. wash) are expected to operate (Collins et al., 1997; Evans et al., 2016). Samples of channel banks were retrieved from the bottom two thirds of the bank profiles to avoid the collection of material more reflective of surface soils and in so doing to help maximize source discrimination. Each sample was a composite of approximately five subsamples taken from within 2 m of the individual sampling site. Within the Semer Water catchment, large landslips have exposed deposits of erodible material which were sampled to the depth of approximately 10 cm after the top 1 cm of surface material was removed to avoid contamination from displaced topsoils. Samples of the channel bed mud deposits in the Woodhill study catchment were retrieved as a grab sample to a depth of approximately 20 cm.

### 3.2 Laboratory analyses for sediment tracers

The source material samples were initially oven dried at 105°C, before being disaggregated using a mechanical pestle and mortar. Samples were then dry sieved to <63 μm through a stainless‐steel mesh before being wet sieved to <25 μm using de‐ionized water. This decision was based on the particle size distribution data of retrieved suspended sediment samples and a preliminary analysis of the tracer – particle size relationships of bulked source samples. The prepared samples were then oven dried at 105°C once more and disaggregated using a pestle and mortar. Differing combinations of colorimetric, radionuclide and geochemical properties were used in the four study catchments. Geochemistry and radionuclides were used in the River Lyne catchment, radionuclide and colorimetric tracers in Semer Water, geochemistry and colorimetric tracers in Blockley Brook and geochemistry in Woodhill Brook.

To quantify colourimetric tracers, the samples were packed into clear polyethene bags and images of them were captured using a Ricoh MP colour scanner. The images were then imported into Gimp 2 photo editing software and the values of reflected red, green and blue were measured on a scale of 0–255 in the RGB colourspace (Pulley & Rowntree, 2016). Radionuclide activities were quantified using Ortec hyper‐pure germanium detectors using the methods of Foster, Boardman, and Keay‐Bright (2007). A mean of 2.7 g of each sample was packed into PTFE sample pots to a depth of 4 cm. Each sample was measured for a minimum of 1 day and the total number of decay counts for each radionuclide was quantified manually using Ortec Gamma Vision software. The measured counts were corrected for detector efficiency and the activities of (mBq g^−1^) of ^137^Cs, ^228^Ac, 40K, ^234^Th, ^235^U and ^212^Pb were calculated. The concentrations of P, K, Ca, Mg, Na, S, Fe, Al, Ti, Zn, Cu, Ni, Cd, Cr, Pb, Mo, Co and Mn were determined using a Perkin Elmer Optima 7300 DV Inductively Coupled Plasma – Optical Emission Spectrometer. Prior to analysis, samples (~0.25 g) were digested using 5 ml of aqua regia. Every 10th sample was repeat analysed to ensure consistency of results and that samples were adequately homogenized during the sample preparation process.

### 3.3 Classification of source groups and virtual mixture creation

Five source group configurations were formed for each study catchment. The first three were based upon a *k*‐means cluster analysis (Pulley, Van Der Waal, Collins, Foster, & Rowntree, 2017; Walling et al., 1993; Walling & Woodward, 1995) containing two, three and four source groups. Maps of these groupings within the study catchments are shown in Figure S1. The two additional source groups were based upon land use and geology, except for the River Lyne, where a uniform geology meant that two different land use based classifications were used, and Woodhill Brook, where limited discrimination between cultivated land and grassland also resulted in the same source groups for geology and land use. Each source sample was initially assigned the land use it was retrieved from during the fieldwork (including channel banks (Lyne, Semer, Woodhill), bed sediment (Woodhill) and land slips (Semer)) and the geology which underlies it (Figure 1). An initial linear discriminant analysis (LDA) was then used to determine which of these initial source groups were likely to be discriminated successfully using the measured tracers. Where source groups were unlikely to be discriminated efficiently, they were combined into a single source group. These combined source groups are shown in Figure 1 and were as follows:

Blockley land use: Grassland, woodland, cultivated.
Blockley geology: Marlstone and mudstone, sandstone, limestone.
Lyne land use 1: Cultivated and grassland, channel banks, woodland.
Lyne land use 2: Cultivated, grassland, channel banks, woodland.
Semer land use: Land slips, channel banks and topsoils, woodland and peat.
Semer geology: Peat, non‐peat sources.
Woodhill land use: Topsoils, bed sediment, channel banks.
Woodhill geology: Topsoils, bed sediment, channel banks.

For each source group classification, virtual mixtures were calculated to be a 100% contribution from each source and equal proportions of all sources producing between 3 and 5 mixtures for each classification. A mixture of a 100% contribution from each source was the source group median value and the equal proportions were the mean of all source group medians. As this method of forming the mixtures may bias the outcomes in favour of a distribution that is formed around the median (25th–75th percentile range) rather than the mean (TMV Normal, Normal), the models were also run using the means as a 100% contribution from each sample (Figure S2).

The mixtures were initially calculated using data from 100% of the retrieved source samples to reflect the most commonly applied assumption used in source fingerprinting studies that the entire catchment is releasing sediment during effective precipitation events. However, as argued above, this assumption does not reflect reality during many rainfall‐runoff events. Accordingly, nine additional sets of mixtures were calculated using a random 90, 80, 70, 60, 50, 40, 30, 20 and 10% subset of the source samples collected for each source group in each study area. For most of the study catchments, 10% of the dataset equated to one sample per source group, although this was also the case for 20 or 30% of the dataset in some source groups. The formation of the virtual mixtures and their source apportionment with the un‐mixing model was repeated a total of 10 times and the mean result was used to interpret model success.

### 3.4 Un‐mixing modelling for sediment source apportionment

An updated version (v1.2) of the SIFT (SedIment Fingerprinting Tool) sediment source fingerprinting software (Pulley & Collins, 2018a) was used for this study; full details are provided in Pulley and Collins (2018b) and a video supporting end‐users can be found at: https://www.youtube.com/watch?v=T8NopA9zgbs&t=84s. For the model runs for each study catchment, three different composite fingerprints (Pulley & Collins, 2018a, 2018b) were formed using a LDA. Each virtual mixture was run through the un‐mixing models with each fingerprint for the 10 sets of virtual mixtures generated. Prior to running the models, all tracers were re‐scaled by dividing by the maximum value in each source group, to ensure the concentration data fell between 0 and 1.

The composition of each of the mixtures was apportioned using an un‐mixing model based upon that developed by Collins et al. (1997) but, critically, using the four different source group tracer distribution methods in a Monte Carlo uncertainty analysis. No corrections for particle size and organic matter content were used as they are not applicable when using virtual mixtures. For the TMV Normal distribution, the tracer values for each source were log transformed and a covariance matrix was formed and a multivariate normal distribution table consisting of 2,000 random values was created from it. This table was sampled for each of the 2,000 Monte Carlo iterations (Batista et al., 2019). Where correlations between tracers were present in the source dataset, they were maintained in the generated distribution (Laceby & Olley, 2015).

The Normal distribution sampled the random numbers according to a normal distribution formed using the mean and standard deviation measured for each source group. There was no removal of potentially outlying samples and correlations between tracers in the source samples were not maintained in the generated numbers. The 25th–75th percentile distributions used the 25th and 75th percentile values of each tracer in each source group. Random values from this inter‐quartile range were sampled for each Monte Carlo iteration. These random values did not follow any specified distribution within the range (e.g. a normal distribution). Correlations between tracers in the source groups were not maintained in the generated random numbers. The Sample Based distribution sampled ~5% of the Monte Carlo iterations from the 0 to 5th percentile measured values of each tracer for each source group, ~5% from the 5th–10th percentile, and so forth (Pulley & Collins, 2018a). In this way, the Monte Carlo iterations roughly followed the tracer distribution of the source material samples retrieved for each individual source group. Where correlations were present between tracers with an *r* ^2^ greater than 0.85, the correlation was also maintained during the random iterations.

### 3.5 Assessment of un‐mixing model performance

Both accuracy and precision of the un‐mixing models using the four distributions were used to assess model success. Model accuracy was quantified as the difference between the median un‐mixing model output and the actual composition of the virtual mixture on the 0–100% contribution from each source scale. Model precision was quantified as the range of uncertainty between the 25th–75th percentile un‐mixing model Monte Carlo outputs on the 0–100% contribution scale. It was also determined if the mean model accuracy exceeded the mean model precision for each of the four study catchments, to identify if the actual model uncertainty was accurately represented in the un‐mixing model outputs. If this was the case, the model output probability density functions were manually examined to determine if the virtual mixture compositions fell outside of the full uncertainty range provided by the models. Finally, one unsuccessfully apportioned site and source group classification was examined in detail. This was to determine how the randomly generated Monte Carlo iterations compared to the retrieved source samples and how the different distributions input into the model result in its specific outputs. The data for additional sites was examined in the same way (but are not presented herein) to ensure that the conclusions made were representative.

## 4 RESULTS

### 4.1 Source discrimination

For all source group classifications, apart from the Four‐Cluster grouping in the River Lyne, source discrimination with the LDA was extremely high, suggesting that the analysed tracers are able to discriminate effectively between the generated source groups (Table 1).

**Table 1:** The percentage of source material samples classified correctly into their respective groups using the three different composite fingerprints for the five source group classifications

Fingerprint	Two cluster	Three cluster	Four cluster	Land use	Geology
Blockley Brook					
1	100	99.9	99.2	98.7	96.8
2	100	99.3	99.2	99.4	96.2
3	100	99.8	98.8	97.7	96.3
River Lyne					
1	98.3	98.2	74.1	97.9	89.4
2	99.7	98.3	73.8	98.9	91.7
3	99.8	98.9	73.9	98.5	91.8
Semer Water					
1	97.5	93.3	94.6	90.8	97.5
2	97.1	93.1	94.5	90.7	97.1
3	97.7	93.1	95.1	90.9	96.8
Woodhill Brook					
1	100	99.9	99.8	100	99.9
2	100	100	99.8	99.9	100
3	100	100	99.7	100	100

A Shapiro–Wilks test for normality was performed for each tracer in each source group and the percentage of source groups that are normally distributed are presented in Table 2. In the Blockley study catchment, most tracers were normally distributed apart from in the two‐cluster classification. In the other catchments, closer to 50% of tracers were normally distributed and for Semer Water, as few as 21% were normally distributed in the two‐cluster classification (Table 2).

**Table 2:** The percentage of source groups * tracers which were normally distributed

Blockley	F1	F2	F3	Lyne	F1	F2	F3
Two‐cluster	40	41	64	Two‐cluster	65	61	55
Three‐cluster	74	81	66	Three‐cluster	61	55	61
Four‐cluster	75	79	83	Four‐cluster	100	100	69
Land use	71	71	75	Land use	67	62	53
Geology	71^a^	71^a^	71^a^	Geology	75	89	71

Semer	F1	F2	F3	Woodhill	F1	F2	F3
Two‐cluster	21^a^	21^a^	19	Two‐cluster	55	50	44
Three‐cluster	46^a^	46^a^	46^a^	Three‐cluster	54	58	58
Four‐cluster	53^a^	53^a^	53^a^	Four‐cluster	54	64	46
Land use	54^a^	54^a^	54^a^	Land use	50^a^	50^a^	50
Geology	33^a^	33^a^	50	Geology	50	50^a^	50^a^

a Fingerprints containing the same tracers as another in that source group classification.

### 4.2 Virtual mixture apportionment results

**Figure 2:**
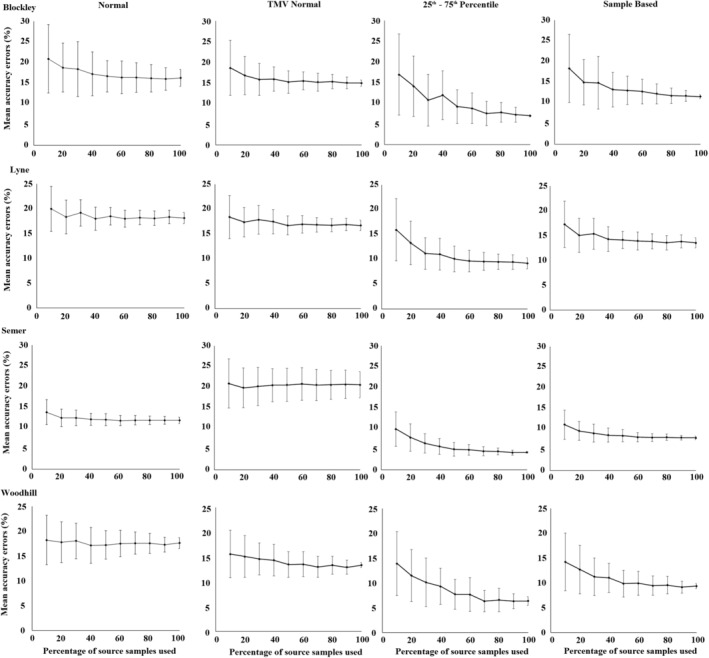
Mean accuracy errors for each site plotted against the percentage of source samples used to form the corresponding virtual mixtures for each study catchment

**Figure 3:**
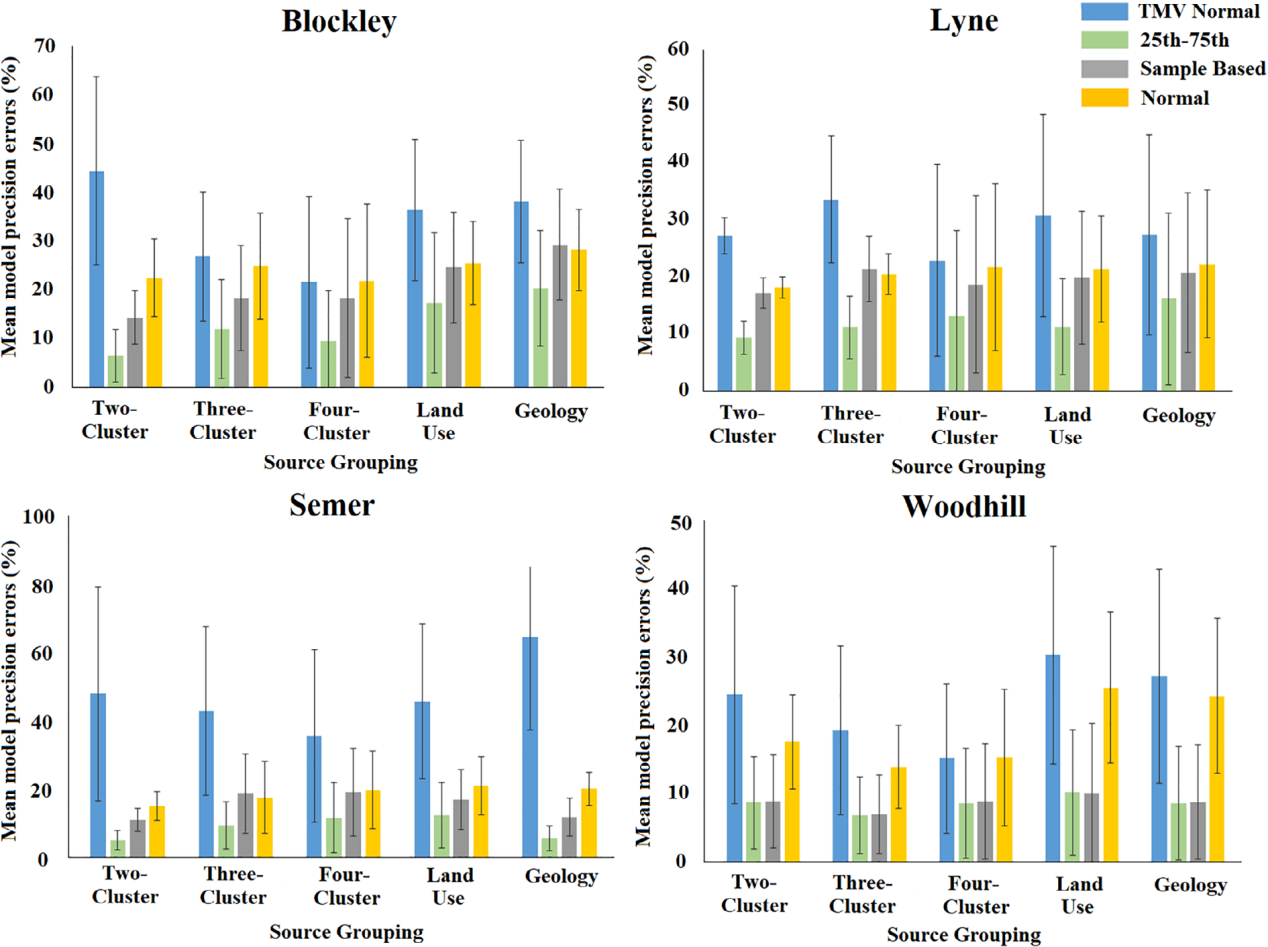
The mean model precision errors, on the 0–100% contribution scale, for all fingerprints and virtual mixtures for the five source group classifications and four study catchments

In all models run, the 25th–75th percentile distribution produced outputs with greater accuracy and precision than the other distributions (Figure 2). Average accuracy errors (i.e. inaccuracy) for all models run were 8.7% for the 25th–75th percentile distribution, 16.9% for the TMV Normal distribution, 16.3% for the Normal distribution and 11.5% for the Sample Based distribution. Average model precision error was expressed as the difference between the 25th and 75th percentile contribution from each source generated by the Monte Carlo uncertainty routine (Figure 3). The mean precision error of the 25th–75th percentile distribution model was 8.5%, which was less than half that of the TMV Normal distribution at 17.3% and Normal distribution at 20.8%, and slightly lower than the Sample Based distribution at 9.3%.

**Figure 4:**
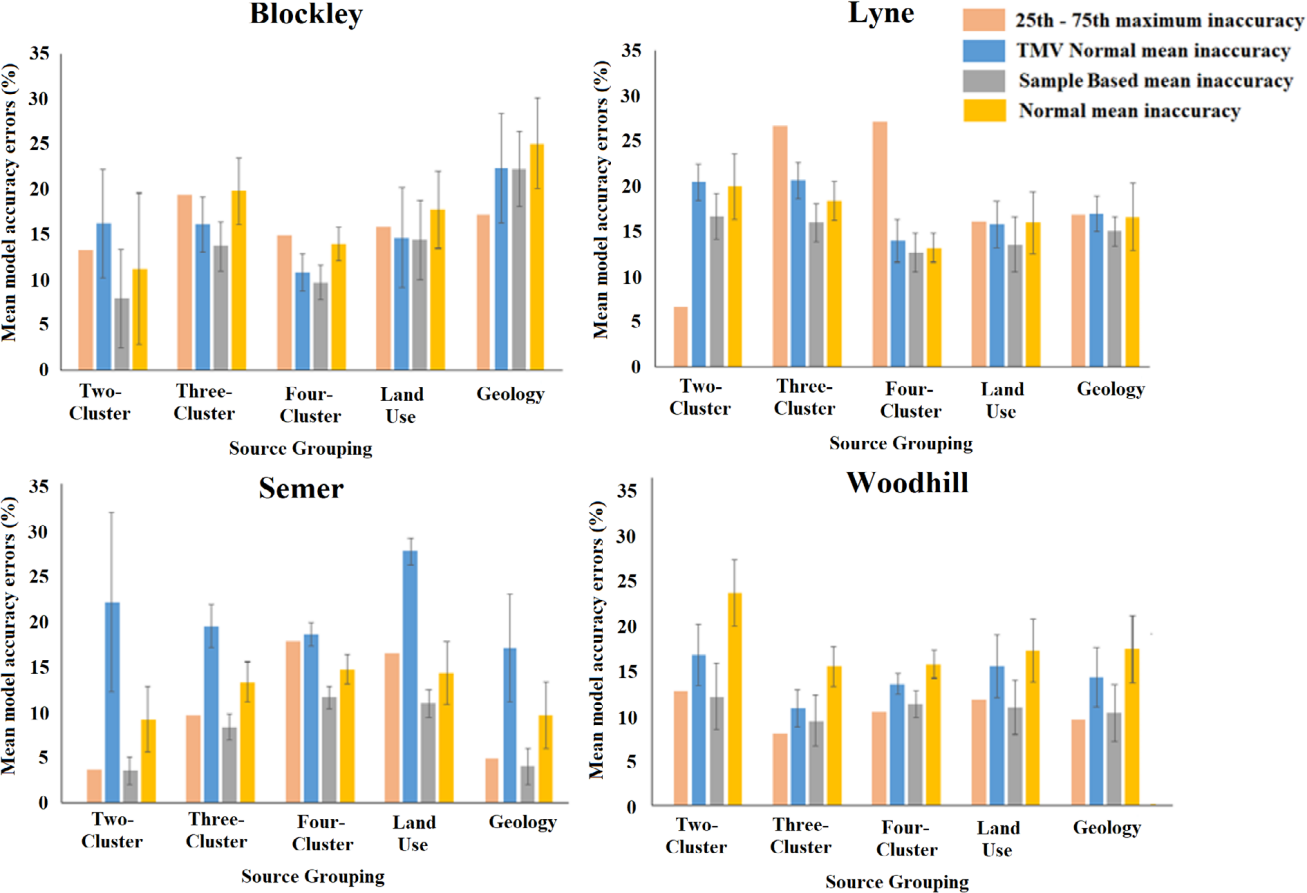
The maximum model accuracy errors, on the 0–100% contribution scale, for the 25th–75th percentile distribution models, and mean model inaccuracy for the Normal and Sample Based distributions

Model inaccuracy increased as a smaller proportion of the source samples were used to form the virtual mixtures. The increase was largest when using the 25th–75th percentile distribution (Figure 2). However, when using this distribution, in only 6 of the 20 source group classifications used, the maximum inaccuracy using 10% of the total source sample dataset was larger than the mean inaccuracy when using the normal type distributions and the entire source sample dataset (Figure 4). The 25th–75th percentile distribution maximum inaccuracy was, however, higher in 14 of the 20 source group classifications when compared to the mean inaccuracy of the Sample Based distribution (Figure 4).

To identify if the actual mixture composition fell outside of the range of uncertainty shown in un‐mixing model outputs, the mean model inaccuracies were compared to the mean model precisions. Mean inaccuracies plus one standard deviation were larger than the mean precision ranges plus one standard deviation in 1 of the 5 source group classifications and 10 virtual mixture sets for the Blockley Sample Based distribution and 1 of the 50 for the 25th–75th percentile distribution. For the 50 mixture sets per site, the following number had a higher inaccuracy than precision: Lyne Sample Based 2, Lyne 25th–75th 5; Semer Sample Based 0, 25th–75th 2; Woodhill Sample Based 9, 25th–75th 6 (Figures 2 and 3). In total, these results represent only 5% of the 200 virtual mixture sets analysed for the 25th‐75th percentile distribution and 6% for the Sample Based distribution. A manual examination of the model probability density functions is required to determine model performance in the few instances when inaccuracy exceeds precision.

### 4.3 Do virtual mixture compositions fall outside of the un‐mixing model output range of uncertainty using the 25th–75th percentile distributions

Having determined that the 25th–75th percentile result is likely to be optimal in the four study catchments, it was assessed if mixtures composed of only a small percentage of the total source sample dataset could generate results where the actual composition of the mixtures fell outside of the range of uncertainty presented in un‐mixing model outputs. The least accurate model results were selected for evaluation where the average inaccuracy was larger than the average precision range in Figures 2 and 3. These were Woodhill land use, Lyne two‐cluster, Lyne‐three cluster and Woodhill three‐cluster.

**Figure 5:**
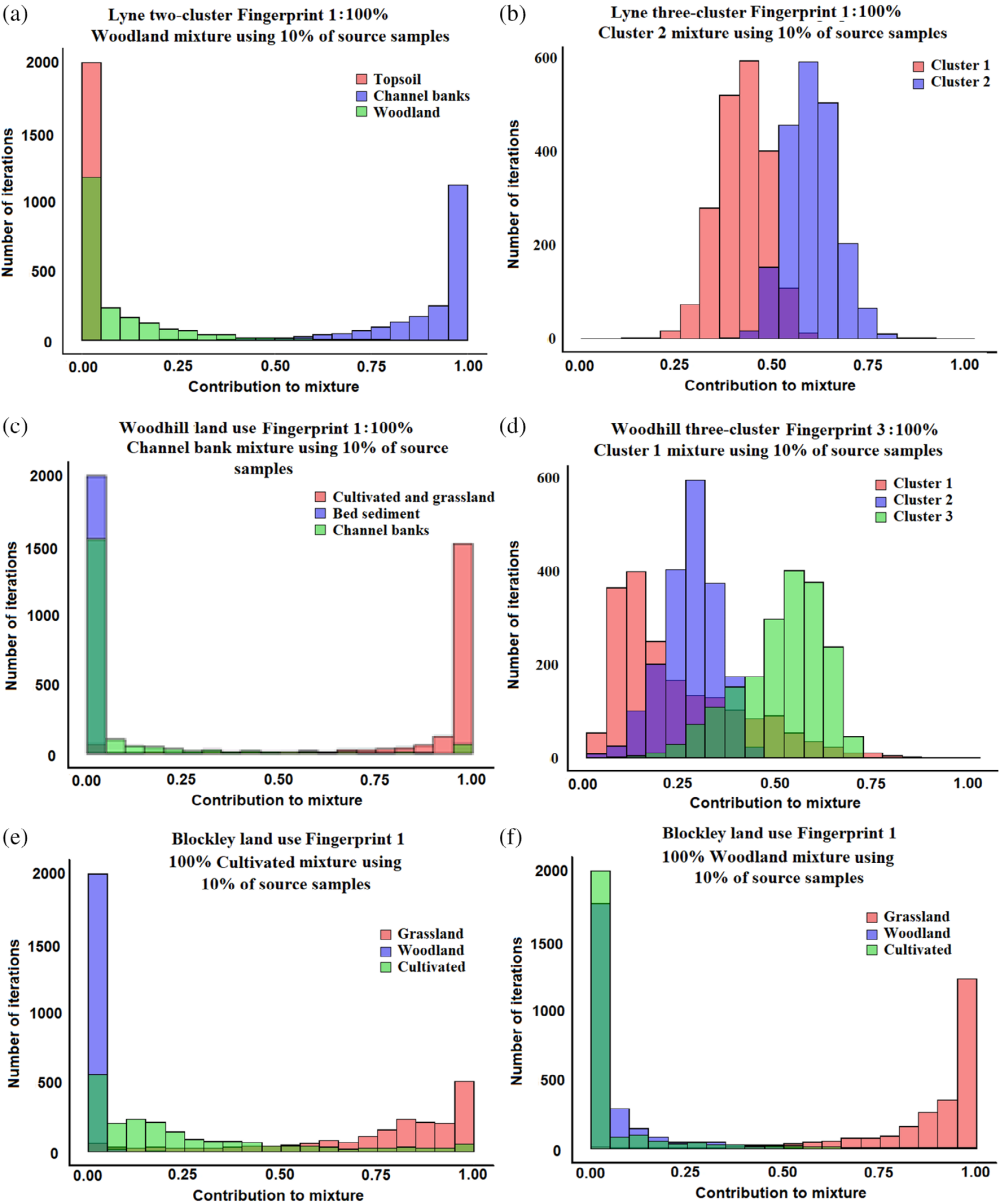
The most inaccurate un‐mixing models when only 10% of available source material samples are used in the virtual mixtures with the 25th–75th percentile distributions

For the Lyne study catchment, two and three cluster Fingerprint 1 and Woodhill three cluster Fingerprint 3 (Figure 5a–c), the mixture composition did fall outside of the range of uncertainty shown in the un‐mixing model outputs. For the Woodhill site (Figure 5d) the least accurate model for land use estimated a 100% contribution from cultivated and grassland topsoils when the mixture was actually a 100% contribution from channel banks. This was likely due to an outlying source sample being randomly selected to form the virtual mixture. In this case, the uncertainty range generated by the model does cover the actual virtual mixture composition. However, it is unlikely that an end user would interpret this result as having high uncertainty and would likely reach an incorrect conclusion that 100% of this sample was composed of cultivated and grassland topsoils. A similar result was found for the Blockley land use Fingerprint 2, when apportioning a 100% contribution from cultivated topsoils, although uncertainty ranges are wider in this case, suggesting that a user may treat this result with more caution (Figure 5e). However, in the same model, the result for a 100% woodland mixture produced an output where the actual mixture composition is outside of the model uncertainty range (Figure 5f).

### 4.4 The generation of random numbers with the Monte Carlo routine and their effect on un‐mixing model accuracy and precision

**Figure 6:**
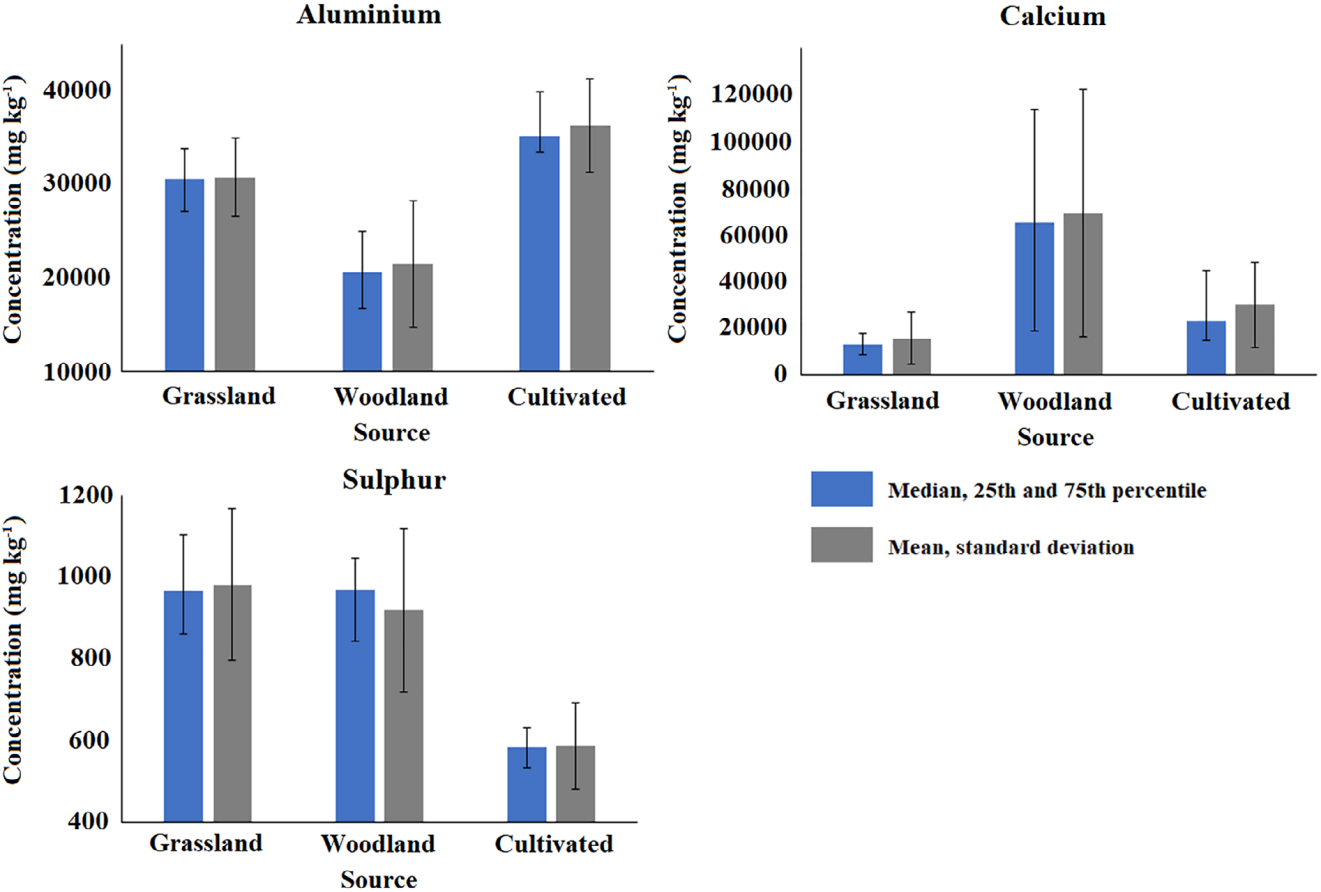
Median, 25th and 75th percentile and mean and standard deviation tracer concentrations in the Blockley Land use Fingerprint 1

To determine why the accuracy and precision of the un‐mixing models using different tracer distributions differ, the Blockley Land use Fingerprint 1 was examined in detail. It was determined how the random values produced during the Monte Carlo routine compared to the measured tracer values of the retrieved source material samples. This fingerprint produced results where the cultivated and woodland sources were not recognized by the un‐mixing model when only 10% of the source sample dataset was used. The composite fingerprint consisted of Al, Ca, Fe, K, Mg, Ni, S and Ti; however, the nature of the discrimination provided by Al, Fe, K, Mg, Ni and Ti was very similar, so only the plot for Al is shown (Figure 6). The mean and median concentrations for each source group were generally similar and any differences between the two averages were far smaller than the differences between the source groups, meaning that the midpoints of the distributions are unlikely to have a significant effect on un‐model results (Figure 6). The standard deviation range was, however, in all cases larger than the 25–75th percentile range. This is particularly important, as only 68% of the generated Monte Carlo iterations would be expected to fall within this range.

**Figure 7:**
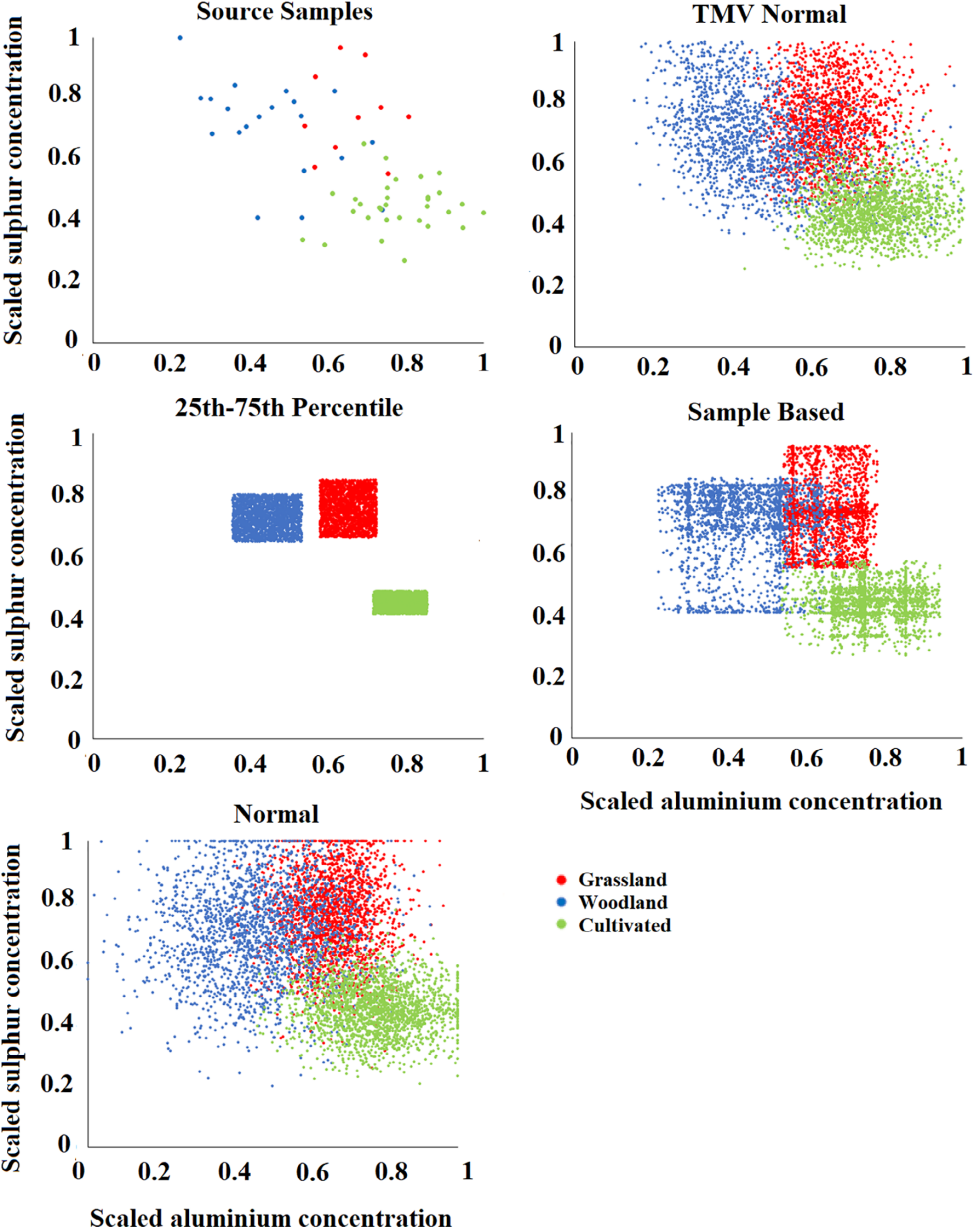
Scaled aluminium and sulphur concentrations in the source samples and generated by the Monte Carlo iterations with the Blockley Land use Fingerprint 1 using the different distributions as un‐mixing model input

**Figure 8:**
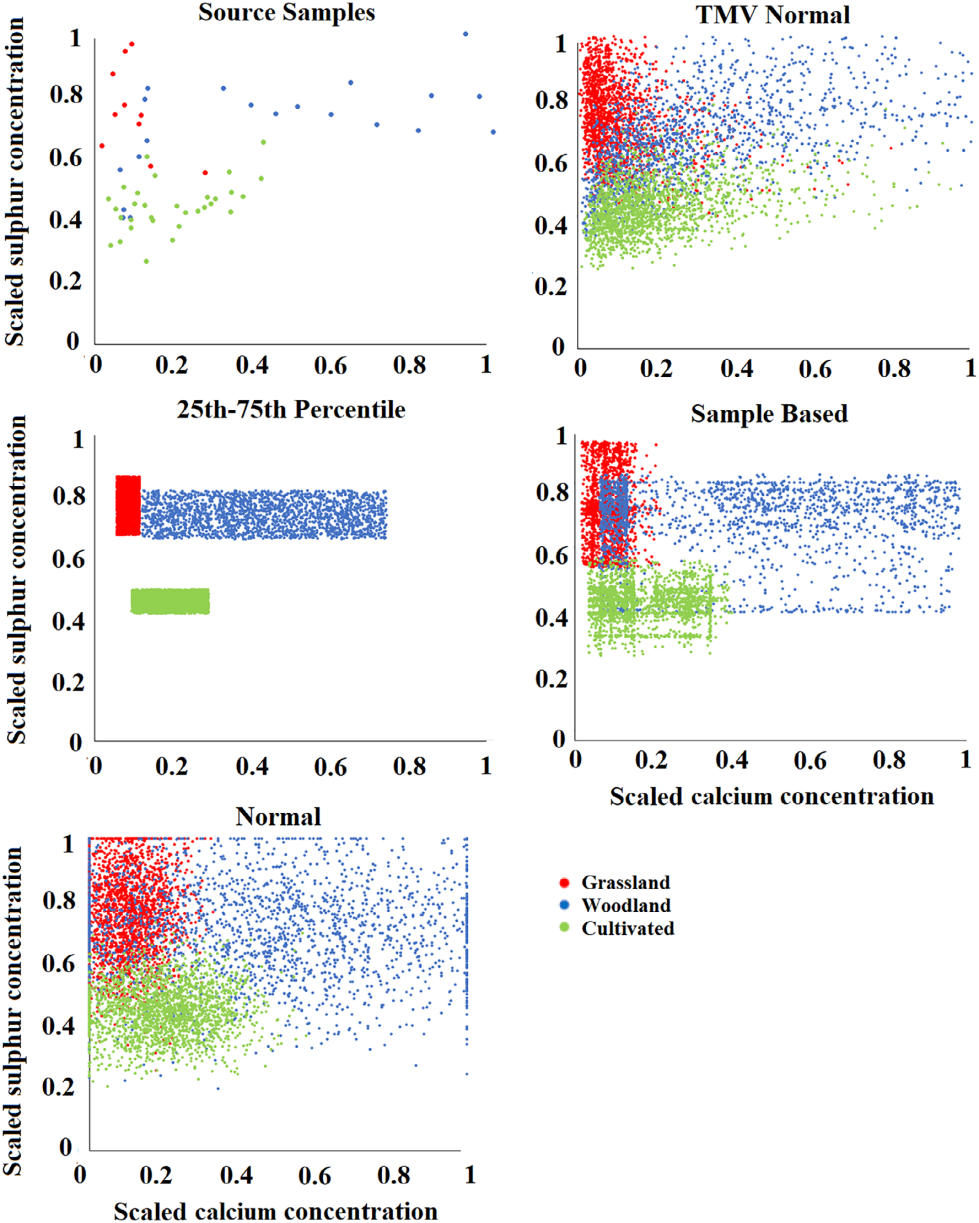
Scaled calcium and sulphur concentrations in the source samples and generated by the Monte Carlo iterations with the Blockley Land use Fingerprint 1 using the different distributions as un‐mixing model input

The scaled concentrations of these three key tracers generated during the Monte Carlo uncertainty routine varied considerably with the different distributions (Figures 7 and 8). The 25th–75th distribution values were clearly truncated when compared to the retrieved source samples, whilst the TMV Normal distribution and Normal distribution produced a broad spread of values which occasionally fell outside of the range of the source groups, especially when the source groups did not follow a regular shape in the plot (e.g. a rectangle). It is of note that the TMV Normal distribution, by maintaining correlations between tracers in the random numbers, produces a distribution more comparable to the retrieved source samples than the Normal distribution. However, the transformed multivariate distribution did produce values which were distant outliers from the range found in the source samples such as for calcium (Figure 8). This is likely a result of imperfect correlations between elements being used to generate the values. As expected, the Sample Based distribution produces values more similar to the source samples than its alternatives; however, a considerable number of values are still produced which fall outside of the range found in the source samples. When considering discrimination between catchment sediment sources, the 25th–75th percentile distribution produced no overlap of values between groups and a smaller within‐source variability (Figures 7 and 8), explaining its more accurate and precise results. However, there is clear scope for an individual retrieved source sample to have tracer values falling outside of the range generated by the Monte Carlo routine. This likely explains why, when only 10% of source samples are used in a virtual mixture model, inaccuracy can be larger than the precision range of the generated model outputs.

**Figure 9:**
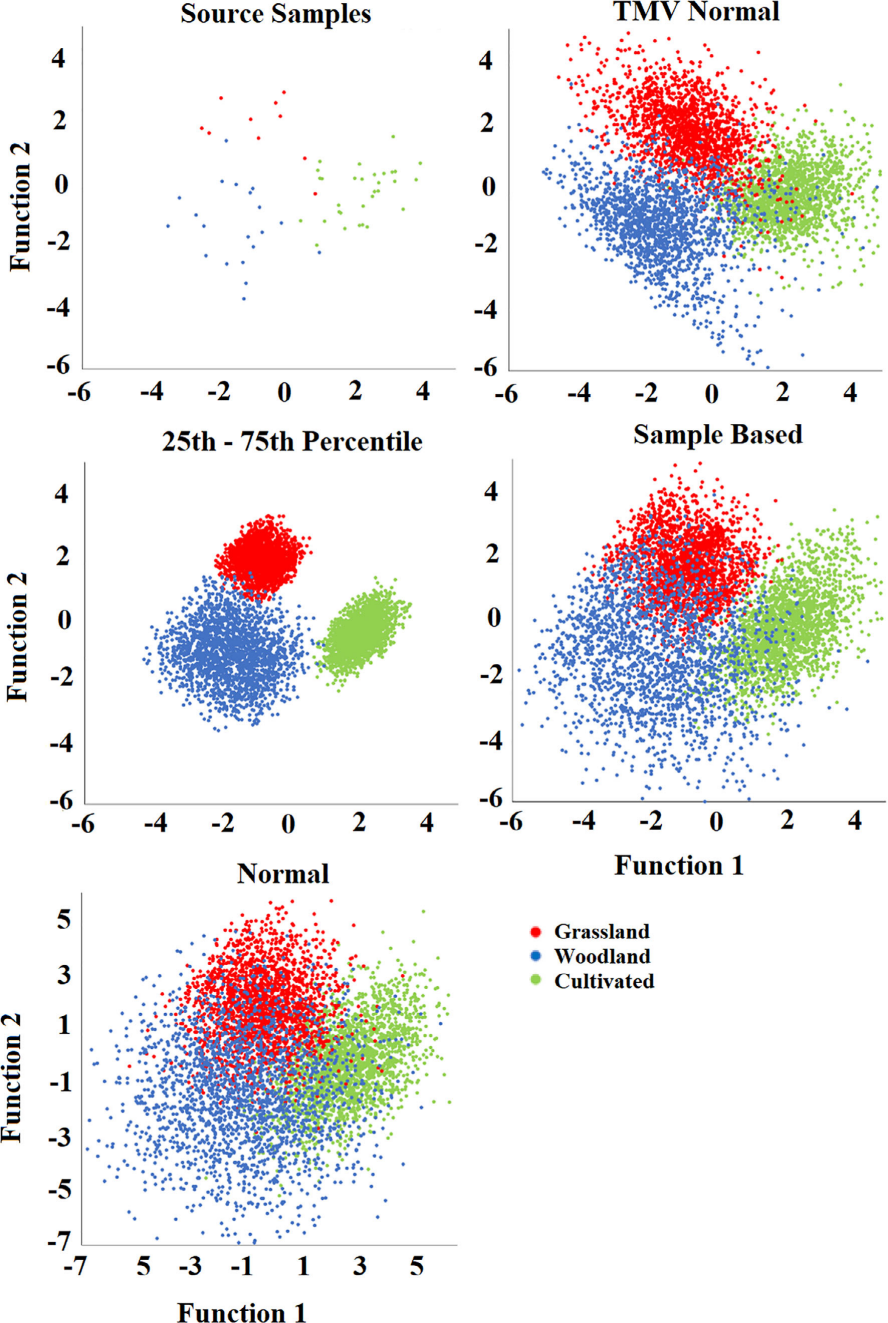
The two largest discriminant functions of the 2,000 Random Monte Carlo iterations generated for the Blockley Land use Fingerprint 1, using the four distributions and for the retrieved source samples

When combining the random values for all tracers in each composite fingerprint into LDA scores, there is a significant difference between the discriminant function scores of the retrieved source samples and those generated by the Monte Carlo routines (Figure 9). In contrast to the individual tracers, the 25th–75th percentile distribution produces a range of values most comparable to the collected source samples. Alternatively, the normal type distributions and Sample Based distribution have a large proportion of iterations falling outside of the range of the source samples. This is likely a result of the combination of random numbers being generated creating values which are not viable in the actual catchment and explains why model accuracy and precision are considerably worse than when using the 25th–75th percentile distribution as un‐mixing model input. Here, it is of note that the 25th–75th distribution cuts out outlying samples such as the two grassland samples that overlap the cultivated group. The removal of such outliers likely explains why in some cases the virtual mixture results fall outside of the range of un‐mixing model uncertainty. It is clear that a single source sample that overlaps source groups can have a large effect on the distributions produced with the normal type and Sample Based distributions. This effect is limited with the 25th–75th percentile distribution is used as un‐mixing model input.

## 5 DISCUSSION

Despite the rapid growth in the international uptake of sediment fingerprinting procedures over the past 20 years (Collins et al., submitted; Walling, 2013), it is noteworthy that some methodological decisions have received far more scrutiny than others (Collins et al., 2017). More specifically, with respect to un‐mixing models, far more attention has focussed on the choice between frequentist or Bayesian frameworks (Davies et al., 2018; Habibi, Gholami, Fathabadi, & Jansen, 2019) and on model structure (Collins, Walling, et al., 2010; Haddadchi et al., 2014; Laceby & Olley, 2015) in conjunction with decisions to include or avoid a variety of corrections or weightings for various factors including particle size or organic matter selectivity (Koiter, Owens, Petticrew, & Lobb, 2018; Smith & Blake, 2014), within‐source spatial variability in tracers (Collins, Zhang, Walling, & Black, 2010; Martinez‐Carreras et al., 2008), tracer discriminatory weightings (Collins, Walling, et al., 2010; Wilkinson et al., 2013), tracer analytical errors or precision (Collins et al., 1997; He & Owens, 1995) or informative priors based on either strategic evidence on maximum source contributions (Collins, Walling, et al., 2010 ) or slope‐to‐channel connectivity (Upadhayay et al., 2020). Robust assessment of the impact of different tracer distributions on the robustness of estimated source proportions has not featured in existing international literature. This is somewhat surprising since the selection of tracer distributions should be seen as a critical decision in the set‐up of un‐mixing models. Some well‐established frameworks adopted robust estimators for the location (median) and scale (Qn, Sn) of tracer distributions some years ago (Collins, Walling, et al., 2010; Collins, Zhang, Walling, et al., 2010) to reduce sensitivity to the risks of bias associated with constructing conventional Normal distributions using the mean and standard deviation and to avoid implicit reliance on the assumption of data symmetry which remains an issue even with well‐known robust scale estimators such as the MAD (Rousseeuw & Croux, 1993). More in‐depth consideration of different distributions for tracers and their implications for un‐mixing model accuracy and precision has hitherto been under‐researched and the majority of studies continue to use a conventional Normal distribution (e.g.Chen et al., 2016; Chen, Fang, Wang, Tong, & Shi, 2017; Evrard et al., 2019; Habibi et al., 2019; Huang et al., 2018; Smith et al., 2018).

For all model configurations at all study sites, the TMV Normal and Normal distributions had both a lower accuracy and precision than the alternatives. The 25th–75th percentile results consistently provided the greatest accuracy and precision for source apportionment estimates assessed using virtual mixtures, with the Sample Based distribution, on average, being slightly more inaccurate and imprecise. Here, however, it was observed that when examining the probability density functions of the un‐mixing model outputs, the differences between the 25th–75th and Sample Based distributions were often appreciable when making a visual comparison of the model predictions using these different input tracer distributions.

The findings of this study reveal a clear advantage to using only the 25th–75th percentile ranges of tracer values as input for un‐mixing models. Whilst in some models, the actual model inaccuracy was greater than the precision range provided by the model results, this was highly infrequent (<6% of models) and occurred primarily when <20% of the source samples contributed sediment to the mixtures. Maximum inaccuracy most often remained lower than the mean inaccuracies with the use of a TMV Normal distribution and Normal distribution. Despite the generally reasonable performance of the Sample Based distribution, it did not show any significant advantages over the use of a 25th–75th percentile distribution and also could produce inaccuracies larger than the corresponding precision range for model outputs.

From the analysis of the numbers generated by the Monte Carlo uncertainty routine used in conjunction with the un‐mixing model, it is apparent that using a truncated distribution, such as the 25th–75th percentile, produces a combined set of random values for all tracers most comparable to the source samples retrieved from the study catchment in question. It may therefore be possible for an even more truncated distribution to yield further improvements in accuracy and precision. However, taking this principal to its furthest extreme would result in a methodology comparable to the earliest sediment fingerprinting studies where only individual tracer means are used to represent source groups and, accordingly, there are no uncertainty ranges generated for model results. Whilst this early method would likely yield a high accuracy if 100% of the retrieved source samples contributed sediment equally, if erosion and sediment delivery are even slightly localized, as is often observed on the ground, the actual sediment provenance would fall outside of the corresponding uncertainty range. Therefore, whilst it will likely be possible to further refine decisions as to the optimum tracer range to input into an un‐mixing model, to avoid producing artificially low uncertainties associated with un‐mixing model results, this study suggests that the 25th–75th percentile range may be a widely applicable range for achieving high accuracy and precision, but, critically, without uncertainty ranges being unrealistically constrained. Additional advantages to the use of the 25th–75th percentile distribution are that outliers will have limited effect on the un‐mixing model outputs and that fewer source samples will likely be required to characterize this range than with the normal type distributions and especially the Sample Based distribution. This may result in a significant reduction in the resource requirements for delivering a robust sediment source fingerprinting study.

Palazón et al. (2015) identified using virtual mixtures that including more tracers in a composite fingerprint produced more accurate results. It is likely that this is a result of minimizing the effects of outliers randomly selected during the Monte Carlo routine as the findings of our study herein suggest that a combination of randomly selected tracers which are at the tail ends of the input normal distributions could produce a combined set of random values that are not viable when compared to the values found in the retrieved source samples. For conventional approaches, it is therefore likely that a greater number of tracers used minimizes the effects of any individual outlying tracer value selected by the un‐mixing model.

Whilst this study performed a robust analysis of the accuracy and precision of un‐mixing model outputs using virtual mixture tests, an important consideration is that this only represents the uncertainty associated with un‐mixing modelling within the sediment source fingerprinting procedure. There are many additional sources of uncertainty associated with source fingerprinting methodologies including those associated with the selection and sampling of potential sources, sample transportation and pre‐treatment and laboratory analyses of tracers. For example, Pulley (2014) calculated an average coefficient of variation of 7.6% associated with the measurement of radionuclide tracers which was in some cases larger than the differences in tracer concentrations between source groups. Collins et al. (2019) showed that temporal variability in δ13C, δ15N, TC and TN soil properties could lead to close to 50% errors in the apportionment of contributions from surface and subsurface sources from a single field. Whilst these large uncertainties are site‐specific, they illustrate that without a robust overall methodology the optimization of un‐mixing models is unlikely to be sufficient by itself to deliver accurate results. This is an emerging area of research and more work is needed to explore and characterize these uncertainties.

Source sampling density is an additional source of uncertainty associated with the tracer distributions input into an un‐mixing model. To accurately represent the tracer distributions within an un‐mixing model, the variability within sediment sources must be understood. Small, Rowan, and Franks (2002) recommended 20 samples per source; however, in small catchments such as those examined in this study, fewer samples may be adequate. Further work is required to determine how many samples are required to form the different distributions which might be used in an un‐mixing model. The uncertainty associated with tracer measurement can potentially have a large effect on model results if analytical error is high and discrimination is poor (Collins & Walling, 2004). Collins, Walling, et al. (2010) and much subsequent research has varied tracer concentrations of the target sediments to random values using summary statistics on the sample data to incorporate this uncertainty into results. Therefore, whilst there are clearly advantages to using a truncated distribution, such as the 25th–75th percentile, it is important to consider other sources of uncertainty and incorporate their assessment into any methodology explicitly. To not do so, risks underestimating the true uncertainty of a source fingerprinting study. It also continues to be important to use independent evidence to verify source apportionment estimates generated using the fingerprinting approach, since very few studies have been able to do this and thereby rely on assessing un‐mixing model performance alone using virtual or artificial mixtures.

## 6 CONCLUSIONS

The results of this study indicate that the use of a 25th–75th percentile distribution in a Monte Carlo uncertainty routine can deliver a significant improvement in both the accuracy and precision of un‐mixing model results, when evaluated using virtual mixtures. The poor performance of the Normal distribution and TMV Normal distribution is clearly of concern as large inaccuracies and a wide range of uncertainty in model outputs can significantly reduce the robustness of a sediment source fingerprinting exercise. However, it has been shown in some studies that inaccuracies can be lower than found here, with distributions based upon a mean and standard deviation indicating it can potentially provide reliable results in some cases (Haddadchi et al., 2015). The effective removal of outliers (Gorman‐Sanisaca, Gellis, & Lorenz, 2017) and use of similar distributions which may output a more truncated range of tracer values may also serve to significantly improve un‐mixing model results when evaluated using mixture tests. On the basis of our findings reported here, it is recommended that users of sediment source fingerprinting procedures trial the use of the 25th–75th percentile distribution alongside alternatives as significant improvements in un‐mixing model performance may be possible. Many current Bayesian approaches utilize a normal type distribution, or a similar alternative, based upon a mean and standard deviation. Therefore, it should be evaluated if the advantages of the Bayesian approach are enough to justify any potential loss in model accuracy or precision due to the use of a potentially sub‐optimal representation of catchment sediment sources within the model. The use of virtual sample mixtures with different un‐mixing model data input structures provides an important methodological step with which to make this assessment. The additional methodological step used here to assess the impact of sediment mobilization and delivery from only a small proportion of the catchment in question can clearly also provide valuable information for a sediment source tracing study. Specifically, the results presented herein indicate that unless sediment delivery was highly localized comprising <20% of the retrieved source samples, overall un‐mixing model accuracy was not significantly higher than when sediment is assumed to be contributed uniformly from the entire sampled catchment. Clearly, the result of this proposed methodological step will reflect several factors including the scale of the study area in question and the concomitant spatio‐temporal variability in rainfall coverage and complexity of slope‐to‐channel connectivity pathways.

## Supporting information


**Figure S1**. Maps of the cluster analysis derived source groups produced for each catchment.
**Figure S2**. Mean model accuracy errors with standard deviation range when virtual mixtures were formed using mean values for each source group rather than medians.
**Table S1**. The number of samples retrieved for each source group, groups in black are classified by land use and groups in blue are classified by geology.

## Data Availability

Research data are not shared.
