# Supplementary material for: The representation of sediment source group tracer distributions in Monte Carlo uncertainty routines for fingerprinting: An analysis of accuracy and precision using data for four contrasting catchments
Source: Hydrol Process. 2020 Mar 10;34(11):2381–400. doi: 10.1002/hyp.13736 (PMC7318149; doi:10.1002/hyp.13736)
Supplement: Supplementary file 1 — Figure S1. Maps of the cluster analysis derived source groups produced for each catchment. Figure S2. Mean model accuracy errors with standard deviation range when virtual mixtures were formed using mean values for each source group rather than medians. Table S1. The number of samples retrieved for each source group, groups in black are classified by land use and groups in blue are classified by geology. [file HYP-34-2381-s001.docx]

**Figure S1: Maps of the cluster analysis derived source groups produced for each catchment.**

**Figure S2: Mean model accuracy errors with standard deviation range when virtual mixtures were formed using mean values for each source group rather than medians.**

**Table S1: The number of samples retrieved for each source group, groups in black are classified by land use and groups in blue are classified by geology.**

| Blockley | Lyne | Semer | | Woodhill |
| --- | --- | --- | --- | --- |
| 12 Grassland | 33 Cultivated topsoils | 15 Channel bank and subsurface | | 11 Grassland |
| 39 Cultivated | 18 Grassland | 17 Woodland | | 11 Channel bed deposits |
|  | 10 Woodland | 28 Peat |  | 13 Channel Banks |
| 17 Sand and mudstones |  | 21 Landslips | | 83 Cultivated topsoils |
| 44 Limestone |  |  |  |  |
| 10 Mudstone |  | 88 Till |  | 7 Chalk |
|  |  | 12 Alluvium | | 5 Sandstone |
|  |  | 36 Limestone | | 70 Mudstone |
|  |  | 28 Peat |  |  |
